# Report of the Pennsylvania Hospital for the Insane for the Year 1844

**Published:** 1845-04

**Authors:** 


3. *Report of the Pennsylvania Hospital for the Insane for the year* 1844. By THOMAS S. KIRKBRIDE, M. D., Physician to the Institution.

1. The Superintendent of the Ohio Asylum commences his report with a tabular view, exhibiting the prominent points in the history of the patients who have been in the Institution during the year. The most interesting part of this table is in reference to the causes of the disease. Of the 214 cases which are given, 78 are attributed to moral, and 85 to physical causes; 20 to constitutional, and to unknown 31. Of the physical causes, 46 are composed of "ill health" and "puerperal;" but it is probable that in many of these cases, the derangement of the health and the puerperal state only predisposed to the disease by increasing the irritability of the nervous system, the real exciting cause being some trouble or affliction which, in a healthy state of the body, would not have seriously affected the mind.

In the report is contained a retrospect of the results of 541 cases that have been received since the opening of the Institution.

Of these, 254 were recent, and 287 chronic cases; 213 of the former and 182 of the latter have been discharged; of the recent, 188 were restored; of the chronic, 55. Died, of the whole number, 58.

The results of treatment during the past year are shown by the following statement:

| | |
|---|---|
| Whole number under care during the year, - - | 216 |
| Average number during the year, - - - - - | 149 |
| Number admitted the present year, - - - | 68 |
| Discharged, - - - - - - - - | 70 |

Of the number discharged, 38 were recent, and 32 chronic; of the former, 34 were restored, 3 were incurable and 1 died; of the latter, 6 were restored, 20 incurable, and 6 died.

"The months of December, January and February," Dr. Awl says, "were particularly severe upon our epileptic patients. Their convulsive paroxysms were increased in number and severity, followed by an extraordinary sinking and prostration of the vital powers, occasioning death in three instances, and an unusual amount of suffering and weakness in all who survived. We observed something of the same kind of suffering with this class of patients two years since, when, as upon the last occasion, it appeared to assume the form of a special endemic amongst the epileptics. All others continued to enjoy their ordinary health. With the last appearance of this peculiar endemic, the weather was unusually mild and open for the season, and the country extensively inundated from continued rains; the thermometer being greatly elevated, and the barometer depressed for a long period of time."

In regard to the curability of insanity the Dr. observes "that one important fact has been steadily confirmed, not only here, but in the experience of every other institution for the insane, that the ratio of success is largely in favour of recent cases, or those in which the disease has been of less duration than one year before the admission of the patient."

The present report sustains the author's reputation for zeal and industry in the cause in which he has embarked.

2. The report of Dr. Ray, consisting of forty pages, after a short exhibit of the number of patients under care, and the results of their treatment during the year, is principally devoted to a communication of his views in relation to the treatment of insanity. As some of the points discussed are important, and the author's remarks thereupon appear extremely judicious, we will endeavour to give such an outline of them as is consistent with the limits of this notice.

From the equal success that has followed the use of medical means entirely different, he concludes that they, strictly speaking, have less to do than some others with the restoration of the insane; that is, that medicine is less effectual in removing the disease, than that combination of influences which constitute what is called moral treatment; and such he believes is the conclusion to which opinions at the present day are gradually, but surely tending. But, it may be asked, if insanity depends upon a disease of the brain, which is now universally admitted, why should it not be equally under the control of medicine as other bodily diseases. To answer this question he does not consider it necessary to establish any difference between this and other affections. The means upon which the intelligent physician relies in chronic disorders, particularly those of a nervous character, are proper diet and exercise, change of air and scene, and useful and agreeable occupation of the mind. In the treatment of insanity, therefore, it is no departure from the ordinary principles of therapeutics to prefer the means which act directly upon the mind, to those which are applied to the corporeal system.

He would not, however, wish to have it imagined that he places no confidence in any kind of medical treatment. On the contrary, he thinks much may be done by means of medicine to prepare the system for the action of more efficient influences.

Considering insanity to be an affection of the brain, with the nature of which we are but imperfectly acquainted, he has thought "that in the present state of our knowledge, the physician's duty consisted in remedying those bodily disorders with which it is generally accompanied. But when these disorders are removed, when the digestive organs have resumed their healthy condition, when the pulse becomes natural, or nearly so, and no unusual degree of heat or dryness remains in the skin, when the excessive nervous excitement has disappeared, and the patient becomes tolerably calm and self-possessed, while reason is still very far from having resumed her lost dominion, then are we obliged to rely almost solely on moral means."

The entire disuse of mechanical restraints, as practised in some foreign institutions, our author does not consider an improvement in the treatment of the insane. That a hospital for such persons can be conducted without them, nobody doubts; but before it can be admitted that the interests of the insane require their total abandonment, it must be proved, either that their use is actually prejudicial, or that their intended object can be better obtained in some other way. His own opinion in relation to them he gives as follows:

"In most cases where they are now used in American hospitals, I have no hesitation in saying that they are far preferable to the vigilance or force of attendants. The object is gained more surely, more effectually, and with far less annoyance to the patient. A mechanical contrivance performs its office steadily, uniformly and thoroughly, and is submitted to as something inevitable. The will and strength of an attendant are capricious and variable in their operation. The strong effort is occasionally relaxed, and the idea of eluding his vigilance or overpowering his strength, is constantly present to the patient's mind. The former is mere inert matter, and excites no feeling, while an attendant constantly present, watching and restricting every movement, is viewed as the author or abettor of his sufferings; his spirit is chafed, and a constant state of irritation is produced."

Notwithstanding his dissent from the opinions of those who have gone to the length of doing away entirely with the use of such restraints, he is willing to admit "that the experiment thus far has strengthened the important truth, that kind words and interesting employment will be found—much oftener than we have been in the habit of believing—a successful substitute for their use."

| | |
|---|---|
| The number of patients in the Hospital Nov. 30, 1843, was | 68 |
| Received since, - - - - - - - - | 83 |
| Discharged during the year, - - - - - - | 75 |
| Remaining in the Hospital Nov. 30, 1844, - - | 76 |

Of the 75 patients discharged, 46 were considered curable* at the time of admission, and 29 incurable. Of the former, 32 recovered, 11 were improved, 1 not improved, and 2 died; of the latter, 7 were improved, 21 not improved, and 1 died.

3. In the Pennsylvania Hospital at the date of the last report, there were 132 patients, since which 153 have been admitted, and 134 have been discharged or died, leaving 151 under care at the close of the year.

Of those discharged during the year 1844, there were

| | |
|---|---|
| Cured, - - - - - - - | 75 |
| Much improved, - - - - | 22 |
| Improved, - - - - - - - - | 16 |
| Stationary, - - - - - - | 9 |
| Died, - - - - - - - | 12—134 |

The report before us contains the statistics of 592 cases of Insanity, the number which have been under care since the opening of the Institution. We select, as most worthy of notice, a table showing the supposed causes of the disease, as far as they could be ascertained, in all the cases that have been admitted into the Hospital. The following synopsis of this table will show the relative frequency of the moral and physical causes which have produced the disease in 362 cases.

| *Physical Causes.* | | *Moral Causes.* | |
|---|---|---|---|
| Ill health of various kinds, | 82 | Loss of property, - | 40 |
| Intemperance, - - | 35 | Grief, loss of friends, &c., | 30 |
| Puerperal state, - | 22 | Religious excitement, | 28 |
| Other physical causes, | 30 | Other moral causes, such as disappointed affections, domestic difficulties, mental anxiety, &c, - | 104 |
| Total, | 160 | Total, | 202 |

*The term curable embraces all those patients, in regard to whom there seemed, when they entered the Hospital, to be the slightest hope of recovery.

In regard to the use of restraining apparatus, Dr. Kirkbride says:

"After a careful review of the 592 cases which have been under care since the opening of this Hospital, I have no hesitation in saying that all might have been treated without the use of any mechanical means of restraint, had we started with such a determination, or felt any pride in making such a declaration. I feel equally confident, however, that in the few cases in which the mild means already referred to have been employed, the effect in some has been to prolong, if not to preserve life—in others, to diminish serious danger, and to shorten extreme violence, and in scarce a single one to have been productive of the slightest injury of any kind."

